# Microwave Synthesis of Copper Phyllosilicates as Effective Catalysts for Hydrogenation of C≡C Bonds

**DOI:** 10.3390/molecules27030988

**Published:** 2022-02-01

**Authors:** Anastasiya Shesterkina, Kseniia Vikanova, Egor Kostyukhin, Anna Strekalova, Elena Shuvalova, Gennady Kapustin, Tapio Salmi

**Affiliations:** 1 Laboratory of Nanochemistry and Ecology, National University of Science and Technology MISiS, 6 Leninsky Prospect, 119049 Moscow, Russia; anna.strelkova1994@mail.ru; 2 N.D. Zelinsky Institute of Organic Chemistry, Russian Academy of Sciences, 47 Leninsky Prospect, 119991 Moscow, Russia; ks.vikanova@gmail.com (K.V.); kostyuhin.egor@gmail.com (E.K.); evshouvalova@yandex.ru (E.S.); gik@ioc.ac.ru (G.K.); 3 Laboratory of Industrial Chemistry and Reaction Engineering, Abo Akademi University, 3 Tuomiokirkontori, FI-20500 Turku, Finland; tapio.salmi@abo.fi

**Keywords:** microwave synthesis, copper phyllosilicate, selective hydrogenation, 1,4-butynediol, 1,4-butenediol

## Abstract

For the first time, the new microwave-assisted method for the synthesis of copper phyllosilicates on a commercial SiO_2_ carrier was developed. The application of microwave synthesis allowed to decrease the synthesis time from 9 to 6 h compared to the traditional DPU method of preparing chrysocolla. The synthesized catalysts were studied by N_2_ adsorption, TEM and XRD methods. Catalysts prepared by microwave method are highly effective in the selective hydrogenation of the С≡С bond in 1,4-butynediol to 1,4-butenediol and 2-phenylethinylaniline with a selectivity of 96.5% and 100% at full conversion for 2 and 0.5 h of the reaction, respectively.

## 1. Introduction

Copper and copper-containing materials are widely used in catalytic processes due to their electronic structure, which allows varying the oxidation state of copper from Cu^3+^ to Cu^0^ [1], high activity in both hydrogenation [2,3] and oxidation [4,5] reactions, and relatively low cost compared to noble metals. Among the diversity of different copper catalytic systems, copper phyllosilicates occupy a special place due to their unique structure. Copper phyllosilicate, or chrysocolla, is a silica-supported copper material with a sandwich structure consisting of octahedra CuO_6_ layers possessed between tetrahedra SiO_4_ layers [6]. In contrast to traditional SiO_2_-supported copper oxide, phyllosilicate has an improved thermal stability, higher copper species dispersion, and larger specific surface area due to its layered structure [7,8,9]. 

The synthesis of such structured materials is not a trivial task. Among the methods for preparing phyllosilicates, it is worth noting an ion exchange [10], an ammonia evaporation [11], and the sol–gel [12] methods. However, the simplest and most convenient method is the traditional thermal hydrolysis of urea, which makes it possible to obtain phyllosilicate particles of small size [13]. The main disadvantage of this technique is the synthesis time: the decomposition of urea in solution proceeds slowly and depends on its concentration in the solution. Wei Di et al. [14] noted that complete urea hydrolysis takes 24 h during 35%Cu/SiO_2_ catalyst synthesis at urea: copper ratio 3:1. Hong Du et al. [15] also pointed out that 18/%Cu/SiO_2_ phyllosilicate preparation by this method at urea: copper ratio 2:1 proceeds within 24 h to achieve full urea thermal decomposition. Krijn P.de Jong and co-authors [16] reported that complete copper phyllosilicate formation occurs for 7 days during urea thermal hydrolysis at urea: Cu ratio 1:1. 

On the other hand, the development of chemical technology requires the use of new, modern approaches to the synthesis of materials. The use of the microwave (MW) irradiation during the synthesis of catalytic systems can significantly accelerate the decomposition of the precursors used subsequently reducing the preparation time, which, in some cases, can also lead to a decrease in the size of the deposited particles in comparison with conventional synthesis methods [17,18,19].

In this work, for the first time, the synthesis of copper phyllosilicates (chrysocolla) with 10% wt. Cu loading based on commercial SiO_2_ support was performed using the microwave-assisted (MW) deposition–precipitation with urea method. The morphological and textural properties of the samples obtained under the microwave irradiation were compared with the samples prepared by the traditional DPU method. The catalytic properties of the synthesized materials were investigated in the selective liquid-phase hydrogenation of unsaturated compounds. 

## 2. Results

### 2.1. Physico-Chemical Properties of Catalysts

Copper-containing catalysts with the copper phyllosilicate structure obtained by thermal deposition–precipitation with urea were previously studied in detail by our group and investigated in the hydrogenation reaction of nitro compounds [20]. Here, for the synthesis of chrysocolla microwave irradiation was used for the decomposition of urea for the first time and the physicochemical and catalytic properties were compared with catalysts obtained by the traditional DPU method in the selective hydrogenation of different unsaturated compounds.

**Figure 1:**
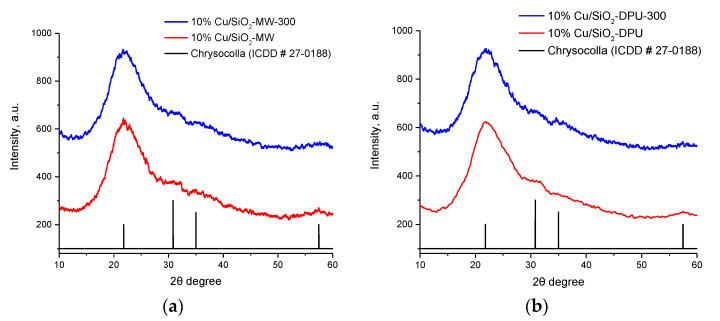
XRD profiles for dry and calcined 10%Cu/SiO_2_ samples obtained by microwave synthesis (**a**) and DPU method (**b**).

The phase composition of all the prepared samples was estimated by X-ray diffraction (XRD) analysis. XRD profiles of samples exhibited a main peak at 2Θ of 22°, which corresponded to commercial amorphous silica, and no obvious peaks corresponding to CuO and Cu_2_O for the calcined catalysts were detected. On both XRD profiles of the dry 10%Cu/SiO_2_-DPU and 10%Cu/SiO_2_-MW (Figure 1) samples, there are broadened reflexes at values of 2Θ at 30.5°, 35.8°, and 56.9°, which ideally agree with the standard reflexes of the (132), (023), and (360) chrysocolla planes. This indicates that copper nanoparticles are strongly dispersed over the surface of the carrier. The dried sample obtained under microwave conditions is slightly more crystallized compared to the DPU catalyst. After calcination at 300 °C in air, the chrysocolla phase was preserved in all samples. Thus, the microwave-assisted synthesis with urea makes it possible to obtain copper-containing catalysts of the chrysocolla structure for 6 h by one-pot synthesis without further calcination. 

**Figure 2:**
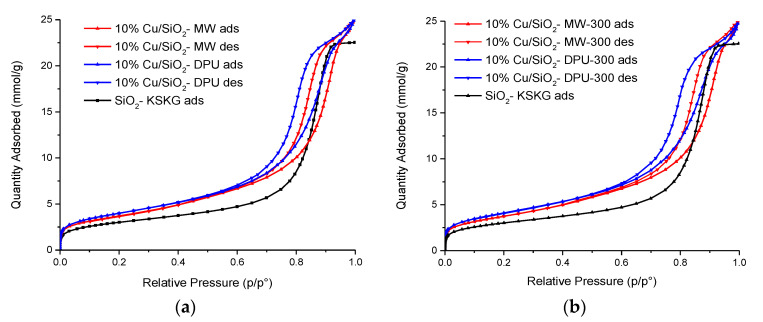
N_2_ adsorption–desorption isotherms of prepared catalysts: dried samples (**a**) and calcined at 300 °C samples (**b**).

**Figure 3:**
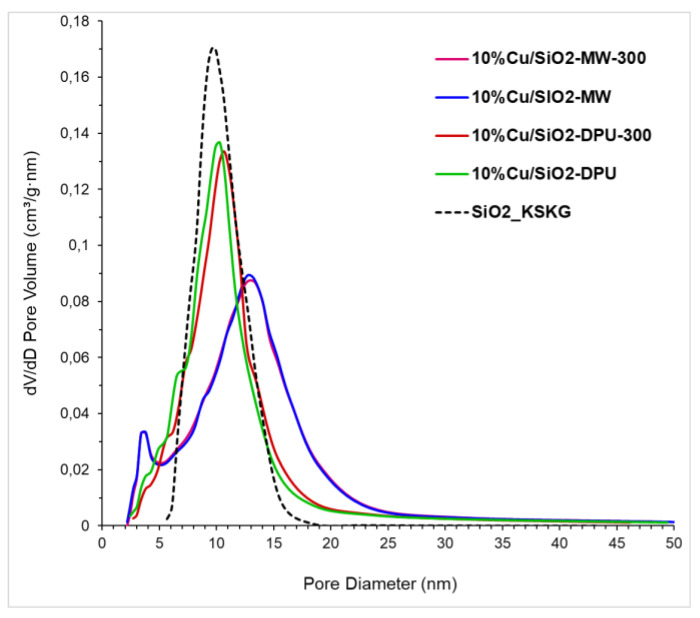
Pore size distributions of the 10%Cu/SiO_2_ catalysts.

The textural properties of the two types of catalysts were presented in Table 1 and Figure 2. The N_2_ adsorption–desorption isotherms of the samples obtained by DPU and microwave-assisted methods with subsequent drying and calcination are shown in Figure 2a,b. The shape of the adsorption curves in all cases belongs to type IV according to the IUPAC classification [21], which indicates the predominance of the mesoporous structure in all samples [22]. Comparison of the synthesized samples isotherms with that of the pure SiO_2_-KSKG has shown the formation of additional narrow mesopores d = 2–6 nm during the copper deposition which are absent in the carrier. The formation of new pores leads to an increase in the specific surface area of the obtained catalysts (Table 1) relative to the SiO_2_ support. The change in the textural characteristics of the synthesized copper-containing samples relative to the initial carrier gives reason to believe that the obtained catalysts have a hierarchical trimodal micro-meso-macro-porous structure. The synthesized catalysts differed in color; the MW samples had a light blue coloration, and the DPU samples were blue-greenish in color (Figure 3). The high specific surface area of copper-containing samples and the color from blue to olive is explained by the presence of copper phyllosilicates, as described in the work [23].

The shape of the hysteresis loops of all catalysts is located between the H1 and H2 types according to the IUPAC classification [24]. This may indicate the presence of various shape pores in the samples: both cylindrical and bottle-shaped [25]. The calculated values of the pore volume and pore size distribution for samples synthesized by the MW method are higher compared to the DPU samples (Figure 3, Table 1). The calcination of samples leads to an increase in micropores volume_s_ and a decrease in surface area relative to dry samples. Thus, it can be noted that the use of the MW-assisted method leads to an increase in volume of micropores compared to the samples obtained by the DPU method and, at the same time, noticeably reduce the synthesis time of the materials.

**Figure 4:**
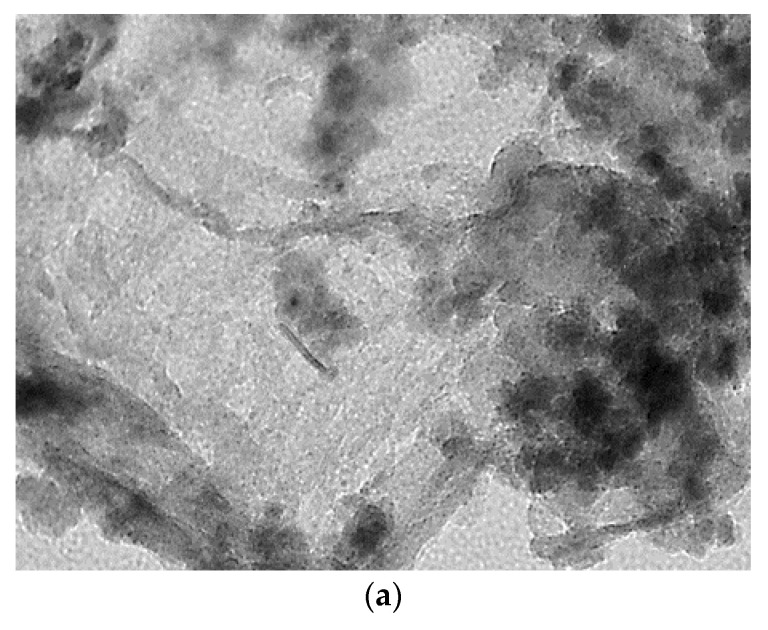

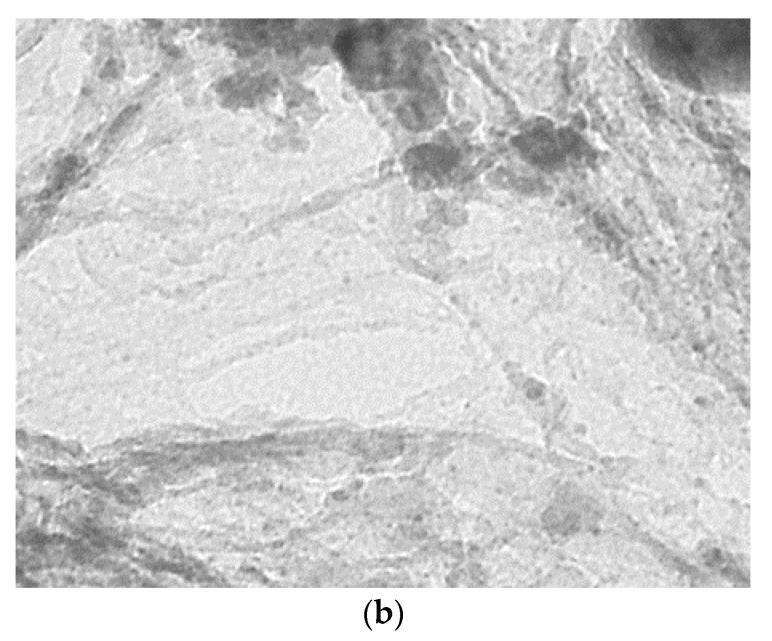
TEM images of calcined 10%Cu/SiO_2_ catalysts obtained by microwave-assisted synthesis (**a**) and DPU method (**b**).

The TEM images of calcined catalysts are presented in Figure 4. Microphotographs of both catalysts show the presence of highly dispersed spherical nanoparticles. In addition, filamentous morphology can be seen, which also confirms the formation of chrysocolla, previously noted by XRD [26]. Statistical results calculated on the 200 particles showed that the sizes of copper particles in 10%Cu/SiO_2_-MW-300 ranged from 4.3 to 5 nm, which was smaller and more uniform than that of the DPU catalyst, where the average particle size was about 7 nm. 

### 2.2. Catalytic Properties in the Selective Hydrogenation of Unsaturated Compounds

The effect of the samples’ preparation method on their catalytic properties was investigated in unsaturated compound hydrogenation reactions. Earlier in our works [27,28], it was shown that, on copper phyllosilicates catalysts obtained by the DPU method, the best catalytical performance in hydrogenation reactions was achieved at temperatures of 150–170 °C and H_2_ pressure up to 2 MPa. 

In the hydrogenation of 1,4-butynediol at temperature of 120 °C, no conversion was detected in the presence of the 10%Cu/SiO_2_-DPU-300 catalyst (Table 2), which might be explained by the fact that, according to TPR-H_2_ studies, the beginning of the reduction of the chrysocolla-like structure begins only above 150 °C. Thus, the optimal reaction temperature for acetylene alcohol hydrogenation was 150 °C.

According to the experimental data presented in Table 2, all synthesized chrysocolla-like samples are catalytically active under selected reaction conditions. However, the catalytic behavior of the obtained Cu/SiO_2_ samples differ somewhat depending on the synthesis method. Comparison of low-percentage samples showed that on a dry 5%Cu/SiO_2_-MW sample, the full conversion of 1,4-butyndiol is achieved in 4 h of reaction with high selectivity (90.2%) for the target 1,4-butenediol. 

Increasing the copper content in the samples to 10 wt.% leads to an increase of the activity of catalysts obtained both in microwave-assisted and thermal DPU methods. However, calcination of catalysts in air contributes to an increase in both the activity and selectivity of the process. The best catalytic properties under the selected reaction conditions were obtained in the presence of a 10%Cu/SiO_2_-MW-300 catalyst, while the full conversion of 1,4-butynediol was achieved in 2 h with selectivity for 1,4-butenediol of 96.5%. The catalyst recyclization have shown the negligible drop of 1,4-butenediol selectivity from 96.5% to 91.2% after the third cycle at full substrate conversion. 

Moreover, the catalyst with the higher catalytic performance (10%Cu/SiO_2_-MW-300) was examined in the reactions of selective hydrogenation of arylacetylenes of various structures (Table 2). Phenylacetylene is a highly reactive compound, and its full conversion was achieved in 0.5 h with a 75.2% styrene selectivity. The relatively low activity and very low selectivity toward stilbene in the diphenylacetylene hydrogenation are associated with its poor solubility in ethanol, which leads to an instant hydrogenation of C≡C to C-C bond. The complete conversion of 2-phenylethinylaniline was achieved in just 0.5 h at a temperature of 160 °C with the 2-(2-phenyl-ethen-1-yl)-aniline selectivity of 100%.

## 3. Materials and Methods

The Cu/SiO_2_ catalysts were obtained by deposition–precipitation with urea by microwave synthesis (MW) and thermal hydrolysis (DPU). The synthesis of 10%Cu/SiO_2_—MW catalyst was carried out in a laboratory microwave system Multiwave Pro (Anton-Paar) under irradiation (2.45 GHz) with urea in four autoclave-type Teflon vessels for 6 h. In a typical synthetic experiment, each vessel was filled with 50 mL of a mixture of decarbonated water and certain volume of 1M Cu(NO_3_)_2_ (Acros Ogranics, 99+%) solution, prepared in advance. Then, fine powder of SiO_2_ (Acros Organics, S_BET_ = 244 m^2^ g^−1^) was added into the solution and stirred. In 15 min, urea (molar ratio urea:copper was equal 6) was added into the obtained colloid solution. Then, vessels filled with the above solution were placed into microwave system and heated up to 93 °C for 6 h. The power of microwave radiation during the synthesis was controlled automatically and was in the range of 70–80 W. The pressure of the system was increased by 4.6 bars, indicating a urea decomposition process. After the synthesis the obtained, the precipitate was separated from the mother liquor by centrifugation (10,000 rpm, 10 min) and washed 2–3 times with 40 mL of decarbonated water. The mother liquor was investigated for the deposition of Cu^2+^ ions onto the support by a qualitative reaction with 1M NH_4_OH solution. The absence of blue coloration confirmed the complete deposition of the precursor onto the support surface. After each wash, the precipitate was separated by centrifugation. The sample was dried under a vacuum on a rotary evaporator at 40 °C and a pressure of 40 mbar for 2 h and then dried at 110 °C in an oven for 5 h. The dry sample was additionally calcined in an air atmosphere at a temperature of 300 °C for 3 h (in a muffle furnace). Catalysts with a copper content of 5wt.% were obtained using a similar technique. Dry microwave samples were marked as хCu/SiO_2_-MW, and the calcined samples were marked as xCu/SiO_2_-MW-300, where x is the mass percentage of Cu (5 or 10 wt.%). 

To compare the catalytic and physical–chemical properties, 5–10% Cu/SiO_2_ catalysts with a chrysocolla structure were prepared by deposition–precipitation of Cu(NO_3_)_2_ on the outer surface of the SiO_2_ support using thermal hydrolysis of urea (DPU). A detailed method of preparation is described in our previously published article [25]. Decarbonized distilled water (46.8 mL), 1 M Cu(NO_3_) solution (3.2 mL), and 1.13 g urea were used for the initial solution for 10%Cu/SiO_2_-DPU sample. However, it should be noted that a longer time of 9 h relative to the MW method is required for the complete deposition of copper ions into the carrier structure by DPU method. The DPU samples were thermally treated according to a similar MW-samples procedure. Dry DPU samples were marked as хCu/SiO_2_-DPU, and the calcined samples were marked as xCu/SiO_2_-DPU-300, where x is the mass percentage of Cu (5 or 10 wt.%).

**Figure 5:**
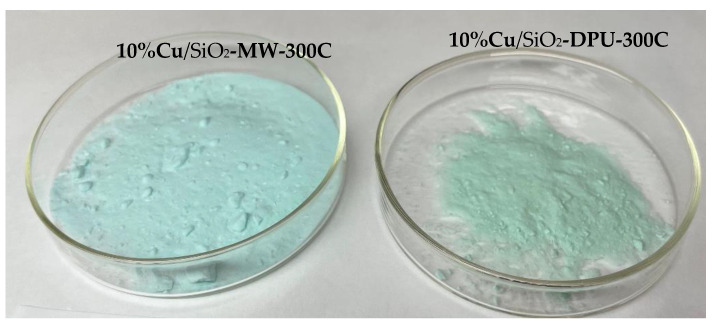
Photos of calcined samples 10%Сu/SiO_2_ obtained by microwave (MW) and thermal (DPU) hydrolysis of urea.

The catalysts obtained by the two methods differed in color; photos of dry and calcined samples are presented on Figure 5. The difference in the color of the samples is probably due to the different volume of micropores.

Chrysocolla-like catalysts were characterized by XRD and N_2_ adsorption–desorption. X-ray diffraction (XRD) were performed after drying and calcination if the samples using an ARL X’TRA diffractometer (Thermo Fisher Scientific) with CuKα radiation (40 kV, 40 mA) with a scanning rate of 1.2° per minute over the scanning range of 10 < 2θ < 60°. ICCD data were used for the identification purpose. 

The N_2_ adsorption–desorption isotherms at 77 K were measured by the Micromeritics ASAP 2020 Plus System. Prior to acquisition of the adsorption isotherm, both dry and calcined samples were degassed for 3 h at 130 and 300 °C under a residual pressure of 0.8 Pa, respectively. The BET method was used to calculate the specific surface area of the sample. Pore size distributions for mesopores were determined by Barret–Joyner–Halenda (BJH) method applied to the desorption isotherms with the Harkins and Jura thickness curve. The total pore volume was evaluated at p/po = 0.99. The cumulative volume at desorption in the BJH method was taken as a mesopore volume. The micropore volume was calculated as the difference between the total pore volume and the mesopore volume. The mesopore-specific surface area was calculated as cumulative at desorption in the BJH method. The micropore size distribution was calculated according to the Horwath–Kawazoe model in assumption of a cylinder shape of the pores.

The microstructure of samples was also studied by a JEM-2100 (JEOL, Tokyo, Japan) transmission electron microscope. Before measurements, the samples were mounted on 3 mm carbon-coated copper grids from a suspension in isopropanol. Images were acquired in the bright-field TEM mode at a 200 kV accelerating voltage.

The catalytic properties of the dry and calcined copper samples were investigated in the selective liquid-phase hydrogenation of unsaturated compounds with molecular hydrogen using a stainless-steel autoclave (100 mL) with a probe-withdrawing valve. The reaction conditions of hydrogenation were as follows: a 0.2 M substrate solution in ethanol (15 mL) with undecane as an internal standard, H_2_ pressure 1 MPa, 150–170 °C, and 1–4 h. The stirring rate was 500 rpm (magnetic stirring). Samples of the reaction mixture were analyzed by GLPC with an internal standard method. 

## 4. Conclusions

In this study, a new microwave-assisted method for copper phyllosilicate on a commercial SiO_2_ carrier synthesis is described. The formation of the chrysocolla phase in MW samples is confirmed by the XRD and TEM results. The catalytic properties of the copper samples strongly depend on their composition and conditions of thermal treatment. The calcined samples are the most active catalysts in selective hydrogenation C≡C bonds. The best catalytic properties were obtained in the presence of a 10%Cu/SiO_2_-MW-300 catalyst, the full conversion of 1,4-butyndiol on which was achieved in 2 h with a selectivity for 1,4-butenediol of 96.5%. Additionally, the 10%Cu/SiO_2_-MW-300 catalyst is highly active and selective in the hydrogenation of arylacetylenes to form a C = C bond with selectivity of 75.2 and 100% to styrene and 2-(2-phenyl-ethen-1-yl)-aniline, respectively. The fast microwave synthesis method is an excellent alternative to traditional methods of synthesis of chrysocolla-like systems, which require a very long synthesis time from 9 h to several days.

## Figures and Tables

**Table 1:** Texture properties of the synthesized samples.

Sample	S_BET_, m^2^/g	V_tot_, cm^3^/g	V_meso_, cm^3^/g	V_micro_ (*t*-Plot), cm^3^/g	D_av_, nm
10%Cu/SiO_2_-MW	303	0.862	0.853	0.0021	1–2, 2–25
10%Cu/SiO_2_-MW-300	299	0.857	0.848	0.0047	1–2, 2–25
10%Cu/SiO_2_-DPU	333	0.857	0.844	0.0017	1–2, 2–25
10%Cu/SiO_2_-DPU-300	323	0.863	0.851	0.0049	1–2, 2–25
SiO_2_-KSKG	244	0.782	0.773	0.0065	6–18

**Table 2:** Catalytic properties of chrysocolla-like Cu/SiO_2_ catalyst samples obtained by MW and DPU methods in the selective hydrogenation of unsaturated compounds.

Substrate	Catalyst	Reaction Temperature, °C	Reaction Time, h	Conversion, %	Selectivity to C=C Bond, %
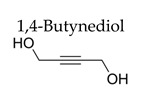	5%Cu/SiO_2_-MW	150	2 4	41.8 100	95.6 90.2
	5%Cu/SiO_2_-DPU	120	0.5	0	0
		150	0.5	17.5	100
			2	40.6	94.1
			4	90.5	89.4
	10%Cu/SiO_2_-MW	150	3	100	96.5
	10%Cu/SiO_2_-MW-300	150	2	100	96.3
	10%Cu/SiO_2_-DPU	150	2 3	63.8 98	95.1 92
	10%Cu/SiO_2_-DPU-300	150	2.5	100	93
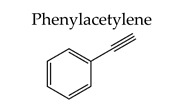	10%Cu/SiO_2_-MW-300	170	0.5	99.5	75.2
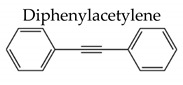	10%Cu/SiO_2_-MW-300	140 160 170	0.5 0.5 0.5 3	3.9 10.2 20 52	35 20.5 24.8 11.9
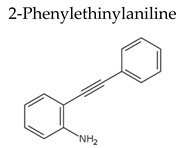	10%Cu/SiO_2_-MW-300	140 160	0.5 0.5	20 99.5	100 100

Reaction conditions: a 0.2 M substrate solution in ethanol (15 mL), H_2_ pressure 1 MPa, 120–170 °C.

## Data Availability

Data is contained within the article.

## Sample Availability

Samples of the compounds are available from the authors.
